# Impact of Carbon Foam Cell Sizes on the Microstructure and Properties of Pressure Infiltrated Magnesium Matrix Composites

**DOI:** 10.3390/ma13245619

**Published:** 2020-12-09

**Authors:** Anita Olszówka-Myalska, Marcin Godzierz, Jerzy Myalski

**Affiliations:** 1 Faculty of Materials Engineering, Silesian University of Technology, 8 Krasińskiego St., 40-019 Katowice, Poland; mgodzierz@cmpw-pan.edu.pl (M.G.); jerzy.myalski@polsl.pl (J.M.); 2 Centre of Polymer and Carbon Materials, Polish Academy of Sciences, M. Curie-Skłodowskiej 34 street, 41-819 Zabrze, Poland

**Keywords:** magnesium matrix composite, open-celled carbon foam, interpenetrating metal/ceramic composites, pressure infiltration

## Abstract

Magnesium-based composites reinforced with open-celled carbon foams (C_of_) of porosity approx. 97 vol % and three cell sizes (20, 45 and 100 ppi) were examined to characterize the influence of foam cell size on the microstructure and properties when pure magnesium and two cast alloys AZ31 and RZ5 were used as matrices. All composites were fabricated by pressure infiltration under the same conditions (temperature, pressure, time). For each matrix composition, two main factors due to the presence of the foam determined the composite microstructure—the efficiency of foam penetration and different conditions of metal crystallization. The lowest porosity was obtained when C_of_45ppi was used and was independent of the applied matrix composition. The metallic component microhardness increased with a decrease in the carbon cell size as well as a decrease in the α-Mg grain size; both of those results should be taken into account during theoretical calculations. Compression and three-point bending strength measurements showed increases as the carbon cell size decreased, but reinforcing effectiveness relative to the matrix material depended on the metal matrix composition. At the fractured surface, different structural effects in the foam and matrix as well as at the interface were observed and depended on the foam geometry, metal composition and mechanical test type. In glassy carbon foam, those effects occurred as cracking across walls, fragmentation, and delamination, while in the matrix, shear bands and intergranular cracking were observed. On the delaminated foam surface, the microareas of a thin oxide layer were detected as well as dispersed phases characteristic for the applied matrix alloys. The accumulation of intermetallic phases was also observed on the metal matrix surface in microareas delaminated from the carbon foams. Mechanical property results indicated that among the tested, open-celled, carbon foams a 45 ppi porosity was the most useful for pressure infiltration and independent of magnesium-based matrix composition.

## 1. Introduction

Ceramic–metal interpenetrating network materials are a group of composite materials that have previously defined component distribution and a defined microstructure that can be obtained by different technologies. Manufacturing solutions for the sintering of ordered ceramic–metal powder mixtures include conventional [1,2] or SHS (self-propagating high temperature synthesis) [3,4] methods as well as infiltration of the porous preforms formed by sintered ceramic particles or short fibers [5,6,7,8,9,10,11,12,13,14,15] and infiltration of open-celled foams of defined geometry and smooth walls on a microscopic scale [15,16,17,18,19,20,21,22,23,24,25,26,27]. The mechanism of ceramic network formation imposes and limits foam characterization features such as cell size, geometry, wall thickness and roughness as well the fraction of open pores; this defines the maximum volume fraction of the second component in interpenetrating network composite materials.

In this work, the problem of open-celled carbon foam (C_of_) selection for a new magnesium matrix composite is described. The literature studies have shown that an application of open-celled ceramic materials of different porosities and cell sizes are proposed for metal matrix composites to improve stiffness, compressive strength and wear resistance [14,15,16,17,20,22,23,24,25]. In these publications the size of cells was characterized variously, i.e., in ppi (pores per inch), mean dimension or mean area of cross-section. SiOC foams with pore sizes of 10, 35 and 50 μm with a porosity of 86 vol % were reported for consolidation with an aluminum matrix by squeeze casting [18]. Gel-cast ceramic foams produced from alumina, mullite and spinel, and characterized of porosities from 15% to 40% have been reported in work [15]. The cell sizes of applied foams ranged from 100–500 μm and they were infiltrated by aluminum, using a pressureless method. Vacuum infiltration was used [19,20] to prepare a SiC foam of porosity 88% and 100 ppi using an aluminum alloy as the matrix. Another fabrication method, pressure infiltration of an aluminum alloy in a vacuum, prepared an Al_2_O_3_ foam of porosity 90% and mean pore size 450 μm [17]. The Si_3_N_4_/Al–Mg system was reported [16] where foams with porosities of 94%, 91%, 88%, 85%, 80% were prepared using pressureless permeation by a metal matrix. For the 3D–SiC/Al–Si–Mg composite [26], the ceramic component (porosity 34%) was applied during a pressureless intrusion. Other efforts [27] reported magnesium matrix composites (AZ31 alloy) interpenetrated by steel, titanium and aluminum reinforcements (porosity 85%) were fabricated using three-dimensional weaving. The authors reported the metal grain size was primarily between 200 and 300 μm and the grain area fraction was not affected by the reinforcement materials.

The manufacturing and properties of the AE44 magnesium alloy reinforced with a SiC−Al_2_O_3_−SiO_2_ ceramic foam were studied in [23]. The interpenetrating phase composite was manufactured by gravity casting at different preheating temperatures of the ceramic foam with porosities between 74.5 and 89%. For glassy carbon foams of porosity 85, 87, 88% and 40, 60, 80 ppi, respectively, results of pressure infiltration experiments for their application in an aluminum matrix have been reported [24,25].

Common to all of these studies was the potential for improving the physical properties; however, optimal foam porosity and cell size conditions have been not determined and data are incomplete to form a general conclusion. In reference [16], of the applied foams, only ones with a porosity of 94% ensured mechanical property increases, while in [15], composites with moderate mean foam cell sizes (200–300 μm) exhibited better properties than composites with smaller or larger cell sizes. In the SiOC-Al system, an increase of properties was reported [18] for cell sizes decreasing from 50 to 10 μm.

Such limited information regarding the potential design of ceramic foam–metal systems stems from two main technological barriers of experimental procedure planning. The fabrication of open-celled ceramic foams with defined open porosity, cell size and morphology (wall thickness, window shapes/sizes, etc.) as well as ensuring enough high mechanical properties are strongly limited by manufacturing techniques. The second crucial technological challenge is components consolidation. As mentioned, pressureless, vacuum and pressure infiltrations can be used in composite manufacturing. Effective and nondestructive movement of the liquid metal in porous ceramics has influenced the chosen infiltration conditions (pressure, temperature, time, atmosphere, wettability) which determines the final composite porosity as well as the bonding character. The nature of that process is quite different from the fabrication of metal–ceramic composite foams, where the contact between liquid metal and the ceramic surface forms an intense metal mixture after which pore formation of metal-based suspension by inert gases occurs [28,29,30,31].

In this work, a magnesium matrix composite with open-celled carbon foam obtained by pressure infiltration is reported; the role of cell size (20, 45 and 100 ppi) for similar foams with an open porosity of ~97 vol %, in microstructure formation and improvement of properties was analyzed. The additional pressure was needed for system consolidation because the wettability of carbon materials by magnesium was unsuitable for spontaneous infiltration as discussed in a previous work from our group focused on C_of_–Mg composite technology [32,33]. The porosity of the applied carbon foam was significantly higher when compared with oxide or carbide foams reported in the literature as components in metal matrix composites; the microstructure of those foams resulted from the nature of carbon material fabrication by pyrolysis and associated with gas emission. The process applied for open-celled carbon foam manufacture [34,35] must ensure the initial porous geometry of the organic precursor foam, that cells with windows formed in resin foam were not degraded or deformed and also the formation of internal defects such as closed pores should be limited. Comparing the C_of_ volume fraction (3%) in these composites, it was lower than conventional composites with microsized reinforcement as carbon fibers (23–70%) [36,37,38,39] or carbon particles (13%) [40] and only a few times higher or similar to those with carbon nanotubes or graphene (0.5–3%) [2,41,42].

The influence of cell size (20, 45, 100 ppi) was analyzed for pure magnesium and two commercial cast magnesium alloys AZ31 and RZ5. The more beneficial foam for the magnesium-based composite was indicated after microstructure characterization and mechanical properties measurements. In addition, the crystallization processes induced in the metal matrix by the presence of carbon cells is examined and discussed.

## 2. Materials and Methods

**Figure 1:**
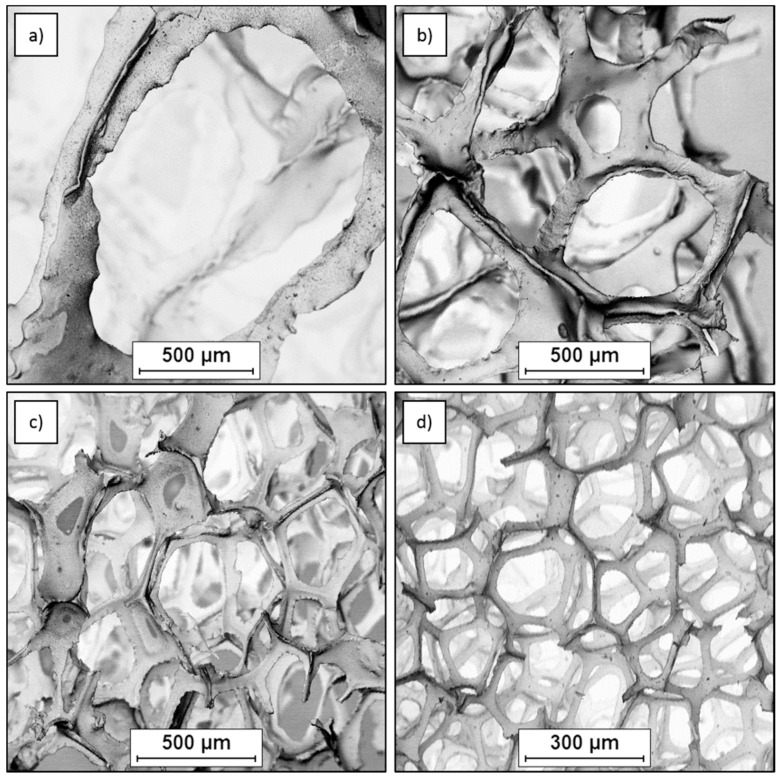
SEM micrographs of different cell size, open-celled carbon foams: (**a**) 10 ppi, (**b**) 20 ppi, (**c**) 45 ppi and (**d**) 100 ppi.

For composite fabrication, the carbon foams obtained at the Silesian University of Technology (Katowice, Poland), Faculty of Materials Science had porosities of ~97% and cells of 20 and 45 ppi were applied as well as a 10 ppi to compare magnesium crystallization effects. As shown in Figure 1a–c they were composed of differently sized spherical cells, and their walls contain the circular windows. The last commercial material C_of_ was called reticulated vitreous carbon (RVC, Duocell, Oakland, CA, USA) with a similar porosity 97%, but cells smaller than 100 ppi and a microstructure as presented in Figure 1d. The cell morphology of the latter was a little different and the windows were huge in comparison with the wall area.

As a metal matrix, technical grade elemental magnesium was chosen which avoided the influence of alloying elements, both in the analysis of the matrix surrounded with continuous solid carbon network crystallization and the processes at the solid–liquid interface. Additionally, two commercial magnesium alloys AZ31 and RZ5 (Table 1) were applied as metallic components; our group and other groups have used these alloys in composites with carbon fibers [32] and glassy carbon particles [36]. Those materials were chosen to use existing knowledge to explain phenomena occurring at the interface of a new interpenetrating network system of foam–metal, as well as to elucidate the role of matrix composition in properties design.

For components consolidation, independently on applied foam and matrix material, a Degussa press (Degussa, Wolfgang bei Hanau, Germany) was utilized and took place under vacuum (2.7 Pa). Components were heated initially in a graphite crucible up to 690 °C and liquid metal under a maximum pressure of 5 MPa was intruded into carbon foams. After 10 min, cooling (over 90 min, rate 7.5 °C/min) under pressure supported with water was applied. Composite samples with 30 mm-diameters 5 mm-thick were obtained.

For determining the open porosity of fabricated materials, the Archimedes method was applied. Their microstructures were characterized by a light microscope (LM, Nikon MA-200, Nikon, Leuben, Belgium), scanning electron microscope (SEM, Hitachi S-4200 (FE) and Hitachi S-3400 N, Thermo Electron Corporation, Waltham, MA, USA) with EDS (Si–Li detector Noran 3500, 130 eV resolution, Thermo Electron Corporation, Waltham, MA, USA). Polished cross-sectioned unetched samples as well as fractured samples were examined. For C_of_–Mg composites with different-sized foams, where the magnesium grain size was characterized, samples were additionally etched with a solution containing: 33 mL H_2_O, 66 mL C_2_H_5_OH, 1 mL HNO_3_, 2 g citric acid, 0.1 picric acid to expose the grain boundaries. For each sample, 15 LM images were registered and measurements were conducted using the Met-Ilo program (author’s original software by Janusz Szala, Silesian University of Technology, Gliwice, Poland) after automatic and manual binary image correction. For AZ31 and RZ5 matrices, that procedure was intentionally skipped due to problems with the correct detection of α-Mg grains with procedures of the automatic image analyze. They were caused by the similarities in the shades of gray for magnesium grain boundaries and other phases seen in magnesium alloys.

For all composites, after 20 measurements, the matrix microhardness levels (HV_0.2_, Duramin-5 microhardness tester, Struers, Copenhagen, Denmark) were determined.

Composite and referential matrix material strengths were determined using an Instron 4469 machine (Instron, Canton, MA, USA). For compressive strength measurements, based on the ASTM E9 standard [43], 5.0 × 5.0 × 9.0 mm^3^ samples were prepared; 5.0 × 4.0 × 30 mm^3^ samples were prepared for the three-point bending tests based on the ASTM C1161 standard [44]. The crosshead testing speed was 5 mm/min under a 5 kN load.

The fractured surfaces were examined with SEM to analyze the decohesion processes of composites and understand the differences associated with applying foams of the same porosity, but different cell sizes, as well as the matrix composition. Observations were focused on the phenomena occurring in foams, matrices, and interface.

## 3. Results and Discussion

### 3.1. Microstructure

**Figure 2:**
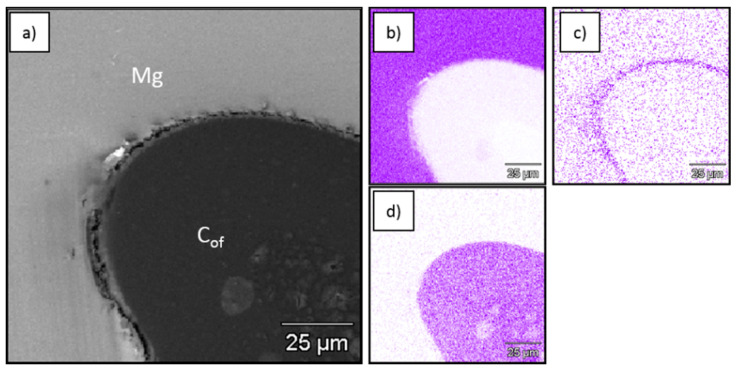
SEM micrograph of C_of_20ppi–Mg composite (**a**) and EDS mapping of (**b**) Mg, (**c**) O and (**d**) C.

**Figure 3:**
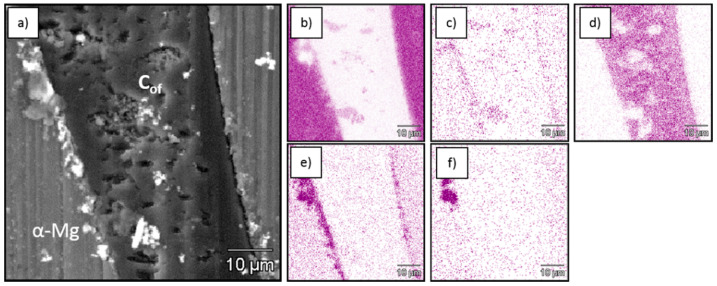
SEM micrograph of C_of_45ppi–AZ31 composite (**a**) and EDS mapping of, (**b**) Mg, (**c**) O, (**d**) C, (**e**) Al and (**f**) Mn.

**Figure 4:**
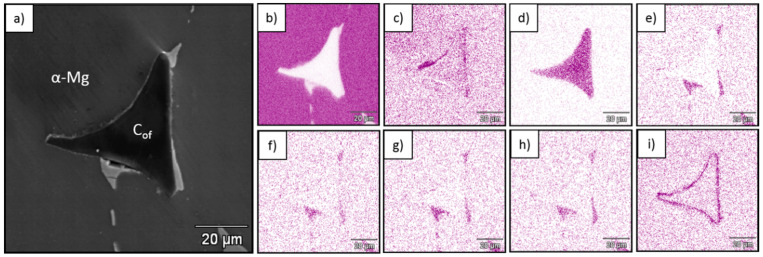
SEM micrograph of C_of_100ppi–RZ5 composite (**a**) and EDS mapping of (**b**) Mg, (**c**) O, (**d**) C, (**e**) Zn, (**f**) La, (**g**) Ce, (**h**) Nd and (**i**) Zr.

Figure 2, Figure 3 and Figure 4 show SEM and EDS images from the obtained composites which revealed continuous bonding between foams and matrices and confirmed well-known structural effects typical for C–Mg systems that depend on matrix composition. The oxygen concentration increased at the foam–metal interface due to the formation of oxide type bonding (Figure 2c, Figure 3c and Figure 4c) [45]. Additionally, the aluminum concentration increased in that region for AZ31, as a result of Mg_17_Al_12_ phase accumulation, oxide phases and Al_4_C_3_ carbide formation. In the case of the RZ5 matrix, except for the magnesium oxide layer enriched with RE (rare earth elements) (Figure 4f–h) the accumulation of Zr (Figure 4i) was observed at the interface, as well as irregular intermetallic phases of RE and Zn (Figure 4g,h).

Open porosity measurement results (Table 2) demonstrated differences dependent on both cell size and matrix composition. Composites with C_of_45ppi had the lowest porosity regardless of the applied matrix composition, but for the same cell size, it was the lowest for a matrix of pure magnesium. The strong porosity increase of (~9%) was characteristic for the C_of_100ppi–RZ5 system and could have been caused by less effective infiltration induced by interphase phenomena, including carbon component destruction by magnesium alloying elements that were more intense for foams with more extended surfaces. That interaction problem has been previously reported for glassy carbon particles—RZ5 composite [40].

**Figure 5:**
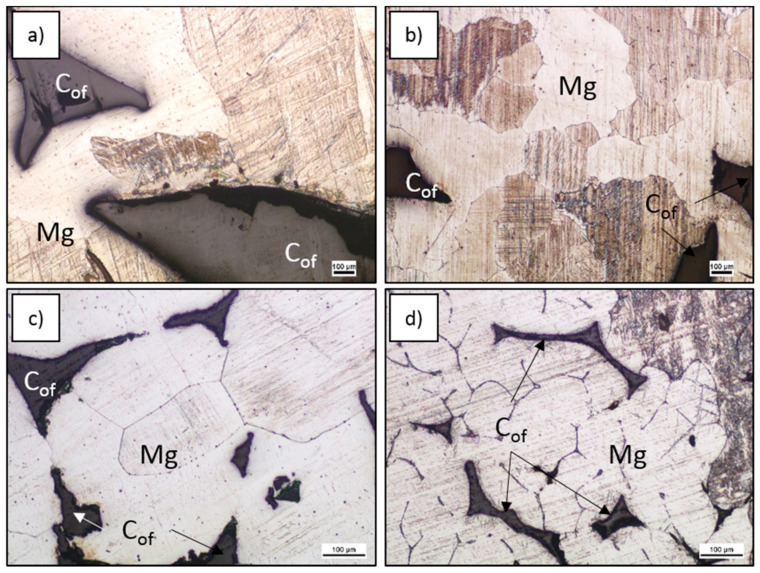
Light microscope (LM) micrographs of etched composites, initial images for quantitative analysis of magnesium grain size (**a**) C_of_10ppi, (**b**) C_of_20ppi, (**c**) C_of_45ppi, (**d**) C_of_100ppi.

The analyses of metal matrix microhardness measurements showed differences dependent on foam ppi, increasing as cell sizes decreased, as well as higher levels than initial cast ingot metals. That effect was not observed for the C_of_–RZ5 composite, where the open porosity was also clearly higher. To explain the microhardness increase, the magnesium grain sizes in C_of_–Mg composites (for completeness with data for C_of_10ppi–Mg composite) were determined (Figure 5 and Table 3) and showed a mean grain area decrease with decreases in foam cell size. This showed the evolution of the matrix microstructure and changes to its properties highlighted a very important problem in theoretical analysis as well as calculating and predicting glassy carbon foam– metal matrix composite properties. The departure of fabricated composites from the rule of components mixture can be caused by changing foam properties (thinner walls, fewer defects), interface microstructure (influence of alloying elements) and by matrix crystallization conditions. The latter factor implies for all calculations, the proper selection of metallic component properties is crucial to estimate the mechanical, thermal and electrical properties of the composite.

### 3.2. Properties

#### 3.2.1. Compressive Strength Test

**Figure 6:**
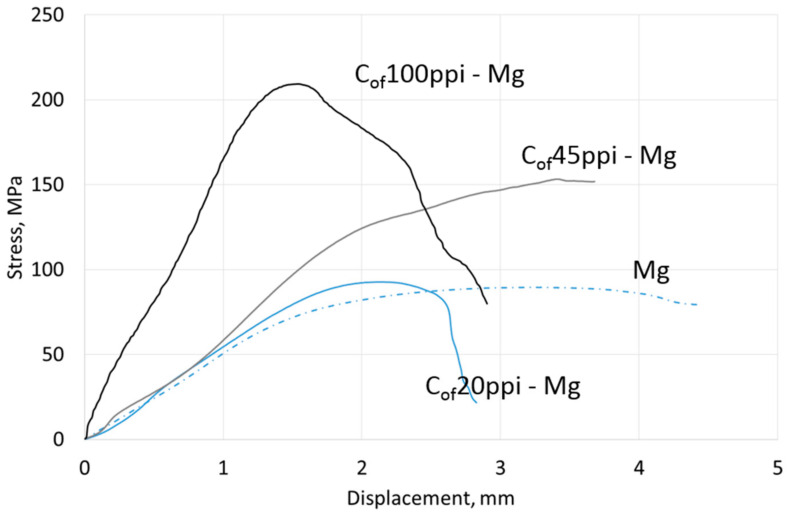
Representative curves obtained in compressive strength tests of Mg matrix composites with C_of_20ppi, C_of_45ppi and C_of_100ppi.

**Figure 7:**
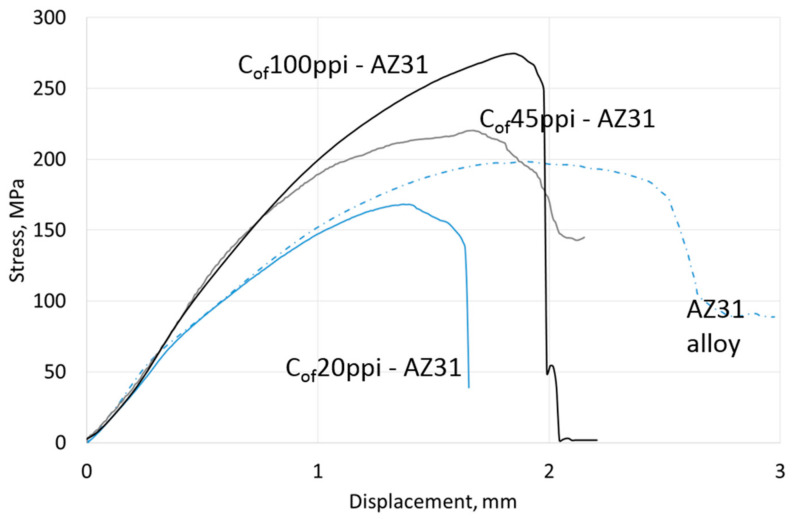
Representative curves obtained in compressive strength tests of AZ31 matrix composites with C_of_20ppi, C_of_45ppi and C_of_100ppi.

**Figure 8:**
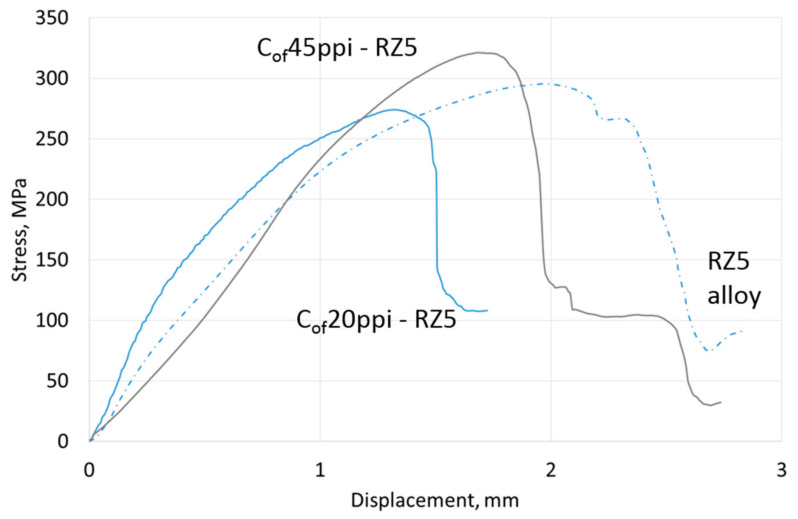
Representative curves obtained in compressive strength tests of RZ5 matrix composites with C_of_20ppi and C_of_45ppi.

Compressive strength results are shown in Table 4, representative curves for materials with different cell size foams and pure Mg, AZ31 and RZ5 matrices are shown in Figure 6, Figure 7 and Figure 8, respectively. They showed a strength increase for all composites with pure Mg matrices, but more intense as the foam cell size decreases. For the AZ31 matrix, the compression strength also increased as the cell sizes changed and were higher than the reference matrix alloy when 45 ppi and 100 ppi foams were used. For the RZ5 composite, only the C_of_45ppi foam showed a strength increase. In general, increases in maximal stress with cell size as well as curve angle increases at the elastic deformation region were revealed; both of which were independent of matrix composition. These results showed the role of carbon foam produced components with higher strengths and varying geometries, and matrix microstructure refining due to crystallization changes.

The C_of_45ppi–RZ5 system is used when comparing values of fabricated carbon foam-magnesium composite strengths, but the most significant increase induced by C_of_ presence was noted for the C_of_100ppi–Mg system. Why changes of the metal matrix involved different strengthening effects with identical foam cell sizes used in the composite, as well as parameters of pressure infiltration are interesting questions. The reason seems to be in phenomena occurring at the components interface.

**Figure 9:**
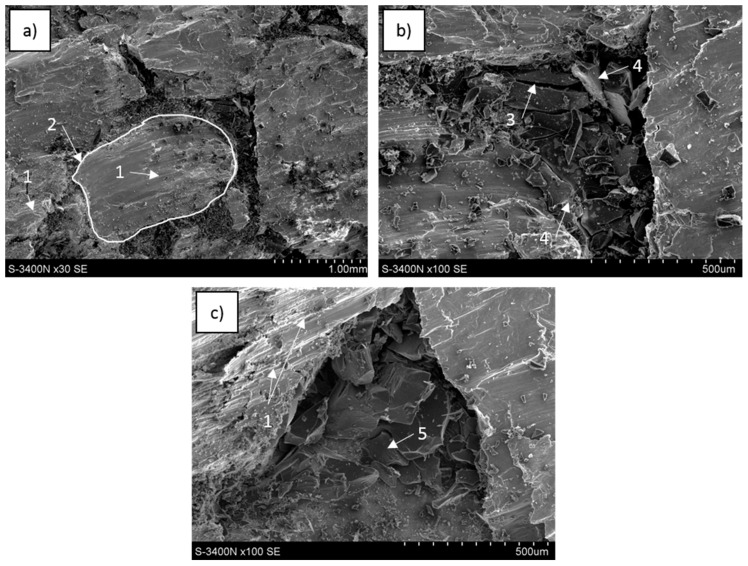
SEM images of C_of_20ppi–Mg composite fractured during compression strength testing: (**a**) metal areas with shear bands (1) surrounded with degraded cells of carbon foam (2), (**b**) cracks in carbon foam (3) and area of carbon (4) coated with an oxide layer formed after contact with liquid magnesium, (**c**) shear bands in magnesium matrix and effect of glassy carbon fragmentation by microcracking (5).

**Figure 10:**
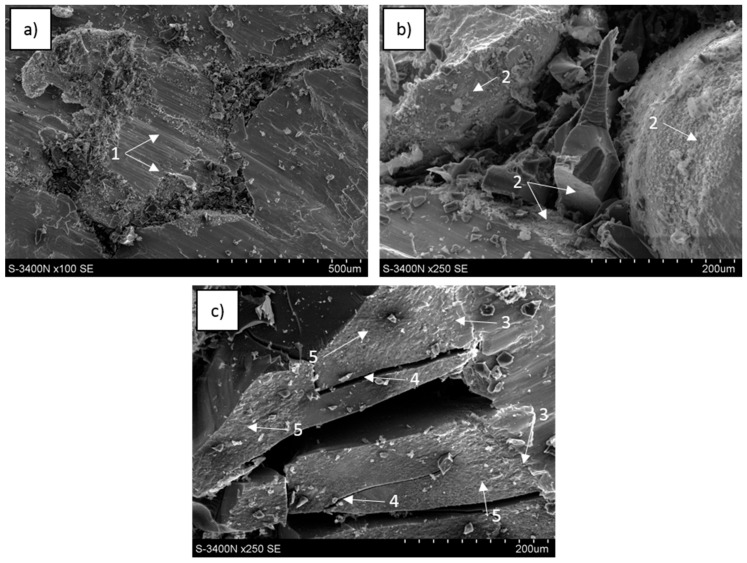
SEM images of C_of_45ppi–AZ31 composite fractured during compression strength testing: (**a**) metal areas with shear bands (1) surrounded with degraded cells of carbon foam, (**b**) particles of refined carbon foam and metal surface after delamination from the foam, both locally coated with a layer of fine phases (2), (**c**) glassy carbon bonded with matrix (3), fragmented by microcracking (4) and locally (5) coated with fine phases formed in contact with liquid matrix and after crystallization.

**Figure 11:**
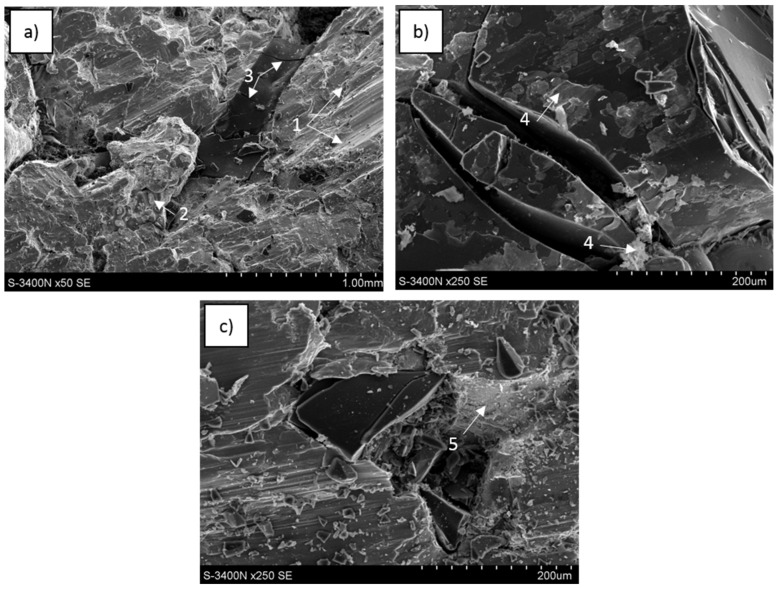
SEM images of C_of_20ppi–RZ5 composite fractured during compression strength testing: (**a**) metal matrix with different oriented shear bands (1) and dendrites (2), carbon foam fractured across the wall (3), (**b**) cracked carbon foam delaminated from the matrix and coated with irregular phases (4), (**c**) cracked and refined foam in the matrix with shear bands, matrix delaminated from foam (5) coated with fine and cracked phases.

Phenomena observed at sample surfaces fractured in compressive tests revealed some characteristic effects in carbon foam, metal matrix and carbon-matrix interface and selected examples are shown in Figure 9, Figure 10 and Figure 11.

In the C_of_–Mg system, when the C_of_20ppi was applied, the carbon network distribution was completely preserved (Figure 9a); however, for C_of_45ppi, the initial carbon position was not clear and no cellular microstructure was visible along the fractured surface on the C_of_100ppi–Mg composite. During composite decohesion, multidirectional cracks in the carbon component were formed and continuous foam transformed into fine particle clusters (Figure 9b,c). As the foam cell sizes decreased, the refinement of the carbon component was more intense. At the metallic surface of the composite, regular and unidirectional shear bands were visible. The pulling out and delamination of carbon foam were not observed, fine carbon pieces coated with a local magnesium oxide layer were detected incidentally and suggested proper bonding between components.

For the C_of_–AZ31 system, the cellular carbon distribution was similar to the pure magnesium composite, visible for C_of_20ppi and incidentally for C_of_45ppi (Figure 10a). Figure 10b,c shows that cracking and refining (less intense) of the foams also occurred. The metallic matrix behavior was also similar—unidirectional shear bands were visible. The main differences were connected to interface microstructure characteristics due to the AZ31 application as decohesion through the foam-matrix interphase region was observed in some fractured composite areas (Figure 10b). Therefore, some foam areas coated with a layer of phases formed and accumulated at the interface during contact with the AZ31 alloy were observed (Figure 10b,c). Moreover, microareas of metal matrix delaminated from the carbon foam (Figure 10b) were coated with very fine phases and confirmed the Mg_17_Al_12_ phase and others shown in AZ31 alloy crystallization at the composite interphase region.

For C_of_–RZ5, in addition to previously mentioned effects such as preservation of carbon reinforcement (C_of_20ppi) initial cellular distribution, many cracks across the foam walls were detected, but the effect of glassy carbon refinement was less intense. At the fractured metallic surface, the multidirectional shear bands were visible as well as dendrites in some microareas (Figure 11a). On the carbon surface, in addition to cracks, a zone of defected interaction products was observed in a region delaminated from the matrix (Figure 11b) and this region was characterized by fine microstructures, many microcracks and an absence of plastic deformation effects (Figure 11c).

It must be mentioned that in compression tests, all reference matrix samples examined were plastically deformed without fracture formation.

Fracture results indicated the reason for differences in reinforcing effects resulted from cell size decrease for the same foam volume fraction in the composite. Ensuring enough low porosity composite results in matrix strength increases, more intense carbon refinement, and higher energy requirements that result in additional, multidimensional cracks.

Weaker or non-existent strengthening effects (Table 4) in systems with AZ31 and RZ5 alloys can be explained by the effects observed at the fractured samples. In composites with applied alloys, decohesion occurred very often at the interface, through the phases formed and/or accumulated during components consolidation though the transformation of carbon foam into fine particles was less intense. This means the interface facilitates crack propagation, thus participation of carbon foam in composite decohesion is less pronounced than for pure magnesium. Moreover, locally registered dendrites in the C_of_20ppi–RZ5 composite explain the high porosity and low strengthening effects in that material.

#### 3.2.2. Three-Point Bending Tests

**Figure 12:**
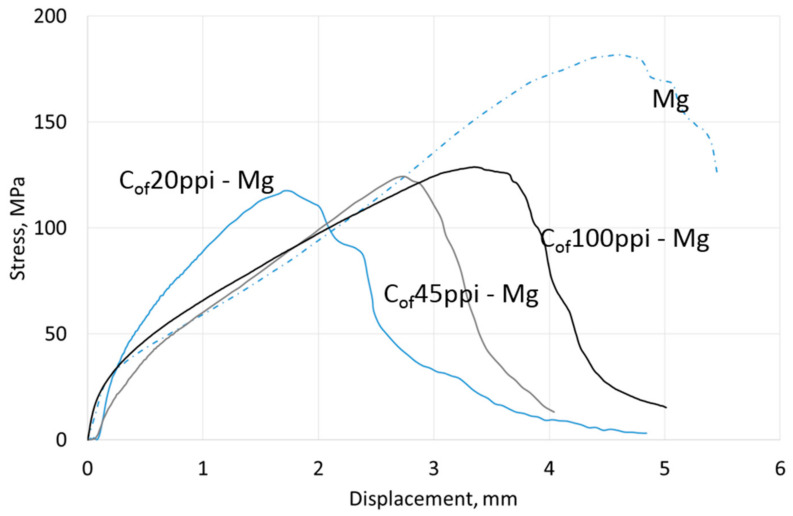
Representative curves obtained from three-point bending tests of Mg matrix composites with C_of_20ppi, C_of_45ppi and C_of_100ppi.

**Figure 13:**
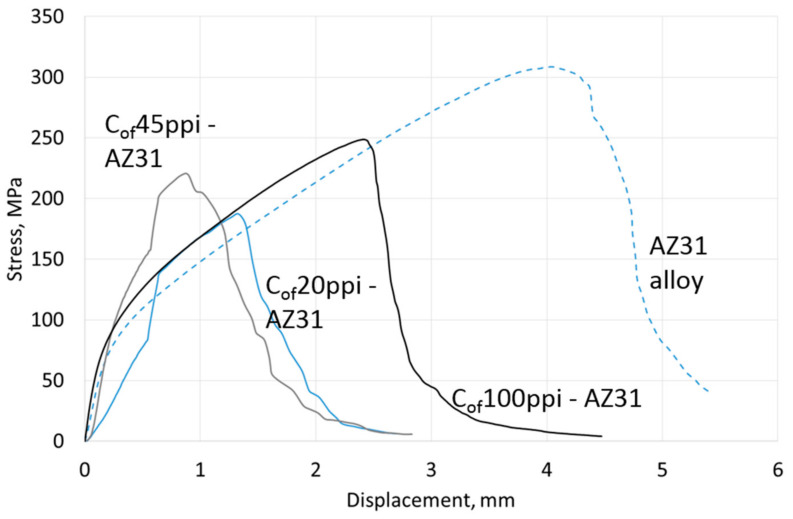
Representative curves obtained from three-point bending tests of AZ31 matrix composites with C_of_20ppi, C_of_45ppi and C_of_100ppi.

**Figure 14:**
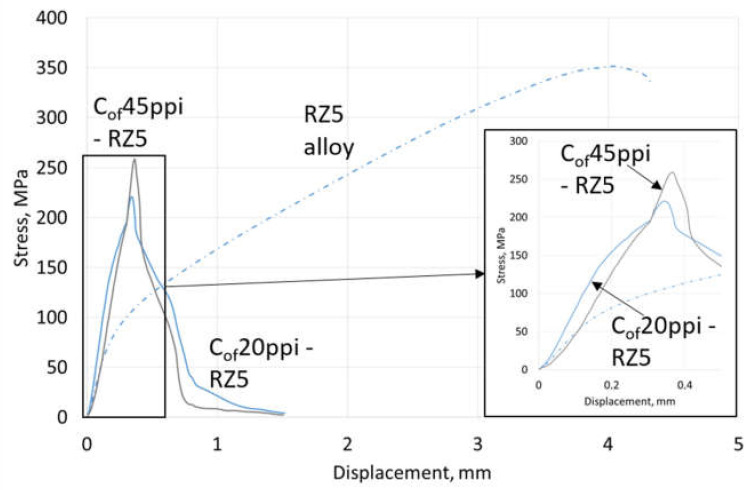
Representative curves obtained from three-point bending tests of RZ5 matrix composites with C_of_20ppi and C_of_45ppi.

Results of three-point bending tests are presented in Table 4; representative curves for materials with different cell size foams and pure Mg, AZ31 and RZ5 matrices are shown in Figure 12, Figure 13 and Figure 14, respectively. They revealed that the bending strength (maximal stress) of composites was always lower than the reference metallic matrix material and is explained by the limited deformation of magnesium due to packed carbon foams cells. The second difference was an increase of the stress vs. displacement curve angle in a region of elastic deformation; that confirmed a stiffness increase induced by the carbon foam which was also observed during compression tests. In addition, a dependence of maximal stress on foam cell size and matrix composition was observed. The bending strength increased as the cell size decreased and increased Mg → AZ31 → RZ5 for composites with the same cell size (Table 4).

**Figure 15:**
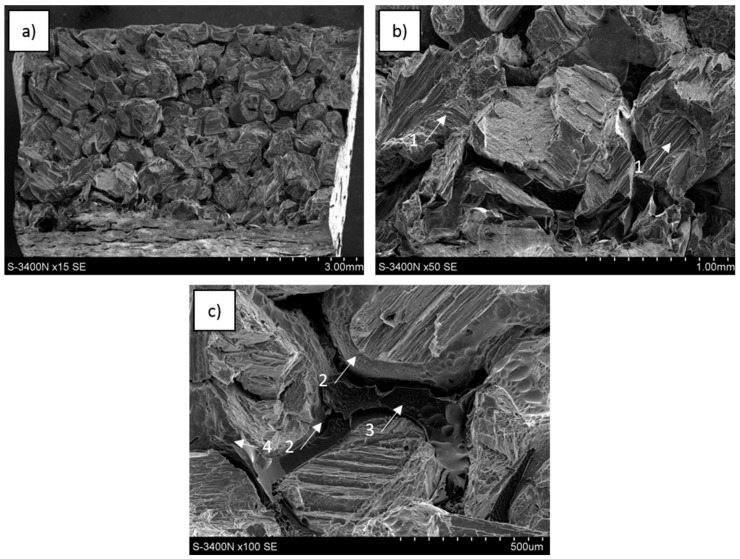
SEM images of C_of_45pp–Mg composite fractured during three-point bending strength testing: (**a**) metal cells surrounded with a glassy carbon network, (**b**) differently oriented shear bands (1) in metal matrix and fragmented foam, (**c**) metal areas with shear bands surrounded with degraded cells of carbon foam, the metal surface after foam delamination (2), foam surface after metal delamination (3), fractured foam surface (4).

**Figure 16:**
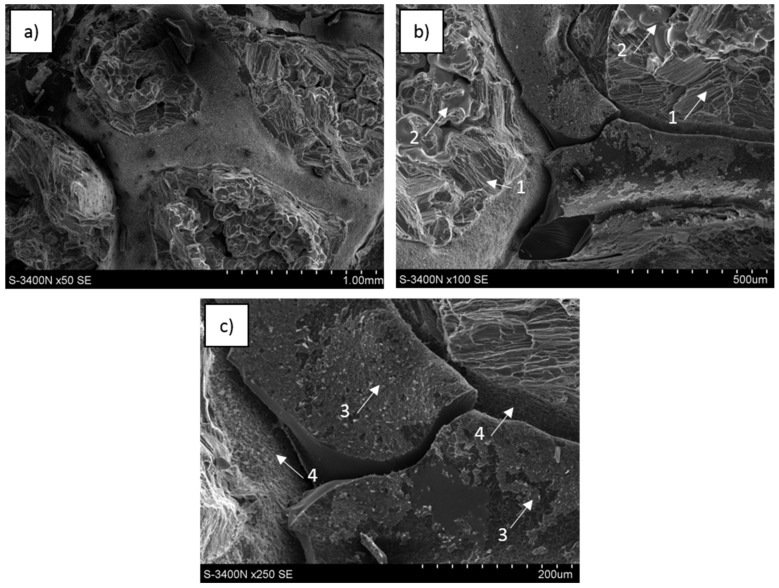
SEM images of C_of_45ppi–AZ31 composite fractured during three-point bending strength testing: (**a**) metal cells surrounded with a fractured glassy carbon network, (**b**) differently oriented shear bands (1) and dendrites (2) in metal matrix and fragmented foam, (**c**) surface of delaminated carbon foam and matrix, thin layer of fine phases on C_of_ surface (3), fine phases on matrix surface (4).

**Figure 17:**
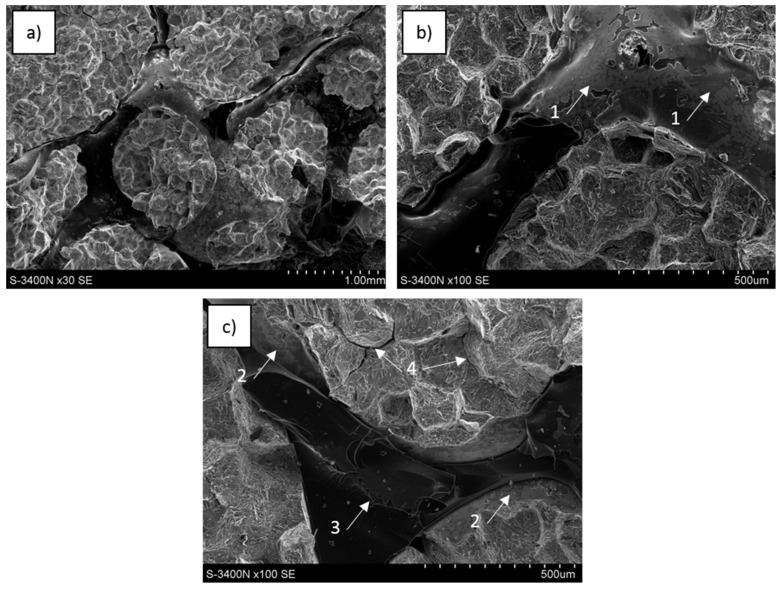
SEM images of C_of_20ppi–RZ5 composite fractured during three-point bending strength testing: (**a**) metal cells surrounded with a fractured glassy carbon network, (**b**) surface of the carbon foam and matrix after delamination, thin layer of fine phases on C_of_ surface (1), (**c**) fine phases on a delaminated matrix (2), C_of_ cracked surface (3) and intergranular cracks in the metal matrix (4).

SEM images of the fractured surfaces from a central region of the sample formed during three-point bending tests are presented in Figure 15, Figure 16 and Figure 17. They revealed similar effects observed during compression tests as well as a few new effects. All reference matrix material samples were also deformed without fracture formation.

In the C_of_–Mg system, the cellular carbon component configuration was preserved (Figure 15a,b), but the foam was cracked, fragmented, partially pulled out and connected with the opposite part of the fractured sample. In the magnesium matrix, shear bands in different orientations in metal areas and closed in single cells were visible (Figure 15b,c). On the glassy carbon surface, two microarea types were observed, one formed by cracking and the other was not degraded though exposed due to matrix delamination and coated with a thin layer of very fine phases. The metal matrix surface after foam delamination was a precise mapping of the carbon reinforcement topography, without plastic deformation effects.

For C_of_–AZ31, the carbon foam network outline was observed at the fracture point (Figure 16a–c) and formed by fractured foam, locally coated with a layer of very fine phases as well as microareas where the foam delaminated during decohesion. The metallic matrix was characterized by differently oriented shear bands and dendrites containing microvoids were locally identified.

Observations of the C_of_–RZ5 system revealed (Figure 17) fragments of carbon foam at the fractured surface. However, decohesion occurred primarily at the matrix-foam interface and phases formed in that composite region were visible as thin layers both on delaminated foam and the matrix surface. Fractured foam surfaces were not often observed. Decohesion of the metal matrix occurred differently than for pure Mg and AZ31; visible intergranular cracks were propagating through the RZ5 alloy and exposed single crystals without parallel shear bands.

## 4. Conclusions

Results from ceramic–metal interpenetrating networks material examination for carbon open-celled—magnesium systems, where carbon foams (~3 vol %) with three different cell sizes, pure magnesium, AZ31 and RZ5 as matrices were used, led to the following conclusions:

1. During applied pressure infiltration, the lowest open porosity of a composite was obtained from C_of_45ppi, regardless of the magnesium-based matrix composition;
2. Changes in matrix microstructure and properties were observed and revealed that as the cell size decreased, the magnesium grain size decreased and the microhardness increased. That phenomenon was caused by metal crystallization in carbon foam cells and cannot be overlooked during theoretical calculations and estimation of composite mechanical, thermal and electric properties. Those observations will need to be considered with well-known issues such as the properties of both the interface and carbon materials;
3. The influence of cell size on compression strength was revealed; it increased as the cell size decreased, but that strengthening depended on the applied magnesium matrix composition. For pure composite with a Mg matrix, that value was always higher than the reference metal, for AZ31 when C_of_45ppi and C_of_100ppi, and for RZ5 when C_of_45ppi were applied;
4. An increase of bending strength as the cell size decreased was noticed during three-point bending tests and was always lower than the matrix reference materials;
5. The angle of stress vs. displacement curves in both tests was higher for C_of_–Mg composites than reference matrix materials and showed that stiffness increased due to the carbon foam application;
6. The best strengthening effect was obtained for the pure magnesium matrix, but using magnesium alloys ensured higher composite strength that pointed to the crucial role of the matrix material on mechanical properties when the foam volume fraction was ~3%;
7. During composite decohesion, in glassy carbon foams, effects occurred, including the initiation and propagation of cracks as well as carbon powder cluster formation; however, those processes were more intense when pure Mg was applied as the matrix material. For AZ31 and RZ5 matrices, crack propagation occurred primarily along the foam–metal interface; at the fracture surface, the surfaces of both delaminated components featured new phases, formed earlier or accumulated during composite processing;
8. At the fractured surfaces of the metal matrix, shear bands were observed and did not depend on foam cell size. One exception was the fracture of the C_of_–RZ5 composite after bending, where intergranular crack propagation occurred, and single metal grains were observed.

The further examinations of the novel composites will be focused on microstructure and properties design, especially of fracture toughness, ability to energy absorption, thermal and electrical properties characterization. The results will show the suspected usefulness of C_of_-Mg composites as materials in automotive and airplane constructions.

## Figures and Tables

**Table 1:** Chemical composition of magnesium cast alloys applied as a matrix component.

Alloy	Chemical Composition, wt %				
	Al	Zn	RE	Zr	Mg
**AZ31**	2.5–3.5	0.7–1.3	-	-	bal.
**RZ5**	-	3.5–5.0	0.8–1.7	0.4–1.0	bal.

RE—rare earth elements.

**Table 2:** Composite porosity results and matrix microhardness measurements.

Material	Open Porosity,%	Matrix Microhardness, HV_0.2_	Microhardness Change in Comparison with Reference Sample,%
reference pure Mg	0.00 ± 0.00	31.19 ± 2.21	-
C_of_20ppi–Mg composite	2.52 ± 0.11	44.82 ± 3.84	+44%
C_of_45ppi–Mg composite	0.46 ± 0.01	51.81 ± 0.88	+66%
C_of_100ppi–Mg composite	1.40 ± 0.05	58.76 ± 1.96	+88%
reference pure AZ31 alloy	0.00 ± 0.00	50.31 ± 3.12	-
C_of_20ppi–AZ31 composite	3.81 ± 0.01	56.03 ± 3.22	+11%
C_of_45ppi–AZ31 composite	1.63 ± 0.02	61.53 ± 2.34	+22%
C_of_100ppi–AZ31 composite	2.45 ± 0.04	69.79 ± 4.08	+39%
reference pure RZ5 alloy	0.00 ± 0.00	69.42 ± 6.66	-
C_of_20ppi–RZ5 composite	3.96 ± 0.08	55.12 ± 6.01	−21%
C_of_45ppi–RZ5 composite	1.33 ± 0.01	61.72 ± 6.72	−12%
C_of_100ppi–RZ5 composite	8.75 ± 0.04	57.02 ± 4.87	−19%

**Table 3:** Results of α-Mg mean grain area measurements in a composite matrix with different C_of_ porosities and reference magnesium ingot.

Magnesium-Based Material	Mean Grain Area, mm^2^
pure Mg	0.704 ± 0.698
C_of_10ppi–Mg composite	0.324 ± 0.309
C_of_20ppi–Mg composite	0.142 ± 0.088
C_of_45ppi–Mg composite	0.109 ± 0.081
C_of_100ppi–Mg composite	0.012 ± 0.009

**Table 4:** Compressive and three-point bending strength measurements.

Material	Compressive Strength, Rs, MPa	Change in Comparison with Matrix,%	Three-Point Bending Strength, Rg, MPa	Change in Comparison with Matrix,%
reference pure Mg	86.1 ± 10.2	-	182.1 ± 5.1	-
C_of_20ppi–Mg composite	91.4 ± 1.9	+6%	118.1 ± 3.2	−35%
C_of_45ppi–Mg composite	121.5 ± 7.4	+41%	124.4 ± 2.8	−32%
C_of_100ppi–Mg composite	199.3 ± 14.2	+131%	129.1 ± 4.9	−29%
reference pure AZ31 alloy	189.2 ± 13.0	-	309.0 ± 7.5	-
C_of_20ppi–AZ31 composite	178.8 ± 14.8	−5%	187.8 ± 4.3	−40%
C_of_45ppi–AZ31 composite	203.0 ± 18.9	+7%	220.4 ± 6.2	−29%
C_of_100ppi–AZ31 composite	260.8 ± 19.5	+38%	249.2 ± 7.3	−19%
reference pure RZ5 alloy	290.6 ± 6.3		351.7 ± 8.6	
C_of_20ppi–RZ5 composite	266.5 ± 10.5	−8%	222.5 ± 8.5	−37%
C_of_45ppi–RZ5 composite	328.1 ± 8.1	+13%	259.6 ± 8.1	−26%
C_of_100ppi–RZ5 composite	*	*	*	*

* high defectiveness of composite excluded proper measurement procedure.
